# Efficacy and safety of drug-coated balloons in chronic total coronary occlusion recanalization: a systematic review and meta-analysis

**DOI:** 10.1186/s12872-024-03993-x

**Published:** 2024-06-26

**Authors:** Yuhao Zhao, Ping Wang, Ze Zheng, Qin Ma, Yuchen Shi, Jinghua Liu

**Affiliations:** Center for Coronary Artery Disease(CCAD), Beijing Anzhen Hospital, Capital Medical University, Beijing Institute of Heart, Lung and Blood Vessel Diseases, Beijing, China

**Keywords:** Chronic total coronary, Percutaneous coronary intervention, Drug-coated balloon, Major advent cardiovascular event, Meta-analysis

## Abstract

**Background:**

With advancements in chronic total coronary occlusion (CTO) recanalization techniques and concepts, the success rate of recanalization has been steadily increasing. However, the current data are too limited to draw any reliable conclusions about the efficacy and safety of drug-coated balloons (DCBs) in CTO percutaneous coronary intervention (PCI). Herein, we conducted a meta-analysis to confirm the efficacy of DCB in CTO PCI.

**Methods:**

We systematically searched PubMed, Web of Science and Embase from inception to July 25, 2023. The primary outcome was major advent cardiovascular events (MACE), including cardiac death, nonfatal myocardial infarction (MI), target lesion revascularization (TLR), and target vessel revascularization (TVR). The follow-up angiographic endpoints were late lumen enlargement (LLE), reocclusion and restenosis.

**Results:**

Five studies with a total of 511 patients were included in the meta-analysis. Across studies, patients were predominantly male (72.9-85.7%) and over fifty years old. The summary estimate rate of MACE was 13.0% (95% CI 10.1%-15.9%, I^2^ = 0%, *p* = 0.428). The summary estimate rates of cardiac death and MI were 2.2% (95% CI 0.7%-3.7%, I^2^ = 0%, *p* = 0.873) and 1.2% (95% CI -0.2-2.6%, I^2^ = 13.7%, *p* = 0.314), respectively. Finally, the pooled incidences of TLR and TVR were 10.1% (95% CI 5.7%-14.5%, I^2^ = 51.7%, *p* = 0.082) and 7.1% (95% CI 3.0%-11.2%, I^2^ = 57.6%, *p* = 0.070), respectively. Finally, the summary estimate rates of LLE, reocclusion and restenosis were 59.4% (95% CI 53.5–65.3%, I^2^ = 0%, *p* = 0.742), 3.3% (95% CI 1.1–5.4%, I^2^ = 0%, *p* = 0.865) and 17.5% (95% CI 12.9–22.0%, I^2^ = 0%, *p* = 0.623), respectively.

**Conclusion:**

Accordingly, DCB has the potential to be used as a treatment for CTO in suitable patients.

**Supplementary Information:**

The online version contains supplementary material available at 10.1186/s12872-024-03993-x.

## Introduction

Chronic total coronary occlusion (CTO) remains one of the biggest challenges faced by cardiologists, accounting for 20% of patients with coronary artery disease detected by coronary angiography [1]. CTO is characterized by thrombolysis in myocardial infarction (TIMI) grade 0, persisting for at least 3 months, as determined by angiography or clinical practice [2]. Successful CTO revascularization can alleviate symptomatic angina, improve cardiac function, and reduce the occurrence of adverse events [3]. Recently, percutaneous coronary intervention (PCI) for CTO has developed rapidly owing to significant improvements in equipment and technology [4].

New-generation drug-eluting stents (DESs), which are the first therapy of choice, are the most commonly used intervention in CTO [5]. Although the long-term prognosis data after DES implantation are favorable, the implantation of multiple stents in the coronary artery may increase the risk of restenosis and thrombosis, impair vasomotor function and distal vessel remodeling after antegrade flow restoration, and exclude other possibilities for future bypass anastomosis in these segments [6, 7].

Drug-coated balloons (DCBs), which were first introduced in 2004, have become an alternative treatment option for DESs, without the limitations of permanent metal implants [8]. Evidences suggest that compared to conventional balloon angioplasty or additional stent implantation with DES, DCBs have a lower incidence of stent restenosis and thrombosis and provide better long-term efficacy for in-stent restenosis (ISR) [9, 10]. Considering the high risk of restenosis or thrombosis and the prevalence of CTO, it seems reasonable to study whether the DCB-only method is a feasible option for treating such lesions. However, only a few small sample sizes and short follow-up studies have evaluated the effectiveness of DCB in treating de novo CTO lesions [11–15]. To shed further light on this issue, we conducted a systematic literature review and meta-analysis to assess the clinical outcomes of DCB treatment for patients with CTO lesions.

## Methods

### Search strategy and study criteria

We searched the electronic databases of PubMed, Web of Science and Embase from inception to July 25, 2023 using the keywords (“chronical total occlusion” OR “coronary chronical total occlusion” OR “chronical total coronary occlusion” OR “CTO) AND (“Drug-coated balloon” OR “balloon” OR “DCB”). No language restrictions were applied, and references of included articles were reviewed to extend the search.

The inclusion criteria were as follows:

1. studies with patients treated by DCB in CTO-PCI.
2. Study sample larger than 20 patients with a follow-up of at least 1 month.
3. Original studies reporting at least one of these outcomes: late lumen enlargement (LLE), target lesion revascularization (TLR), target vessel revascularization (TVR), myocardial infarction (MI) and cardiac death.
4. Articles written in English.

Duplicate reports, comments, case reports, and studies with insufficient information were excluded. This Systematic Review is registered with INPLASY202460026.

### Study selection and study endpoints

The quality of the included studies was assessed using the Newcastle-Ottawa Scale. The evaluation framework is divided into 3 areas: The first part of the method is a five-score rating system that evaluates the selection of study groups in each study. The second section evaluates the comparability of study with the likelihood of gaining two scores. The final part of the tool evaluates the outcomes. Subsequently, studies that scored > 6 scores out of 10 were considered as high quality, 5 or 6 out of 10 were considered as good quality, and less than 5 were considered as poor quality. There were no studies excluded due to poor quality (Supplementary Table 1).

Two independent investigators (ZYH and WP) screened all titles and abstracts identified in our literature search. Divergences were resolved by consensus. All available information, including the number of study patients, year of publication, baseline characteristics and major advent cardiovascular events (MACE), was recorded using Microsoft Excel.

The study endpoints were cumulative MACE, including all-cause death, nonfatal MI, TLR, and TVR. The follow-up angiographic endpoints were LLE, reocclusion and restenosis.

### Statistical analysis

Results were analyzed using a random effects model or a fixed effects model in cases of significant heterogeneity between studies. The I^2^ statistic was used to quantify heterogeneity between studies. If the results were non-heterogeneous (I^2^ < 50%), a fixed effects model was used, while if the results were heterogeneous (I^2^ > 50%), a random effects model was used. In addition, 25%, 50%, and 75% indicated low, moderate, and high heterogeneity, respectively. All analyses were performed using Review Manager version 5.4.1 and Stata software version 14.0. A *P-*value *<* 0.05 was considered statistically significant. Funnel plots were not used to evaluate publication bias due to the inclusion of fewer than 10 studies in the meta-analysis.

## Results

### Research selection and characteristics of the studies

**Fig. 1:**
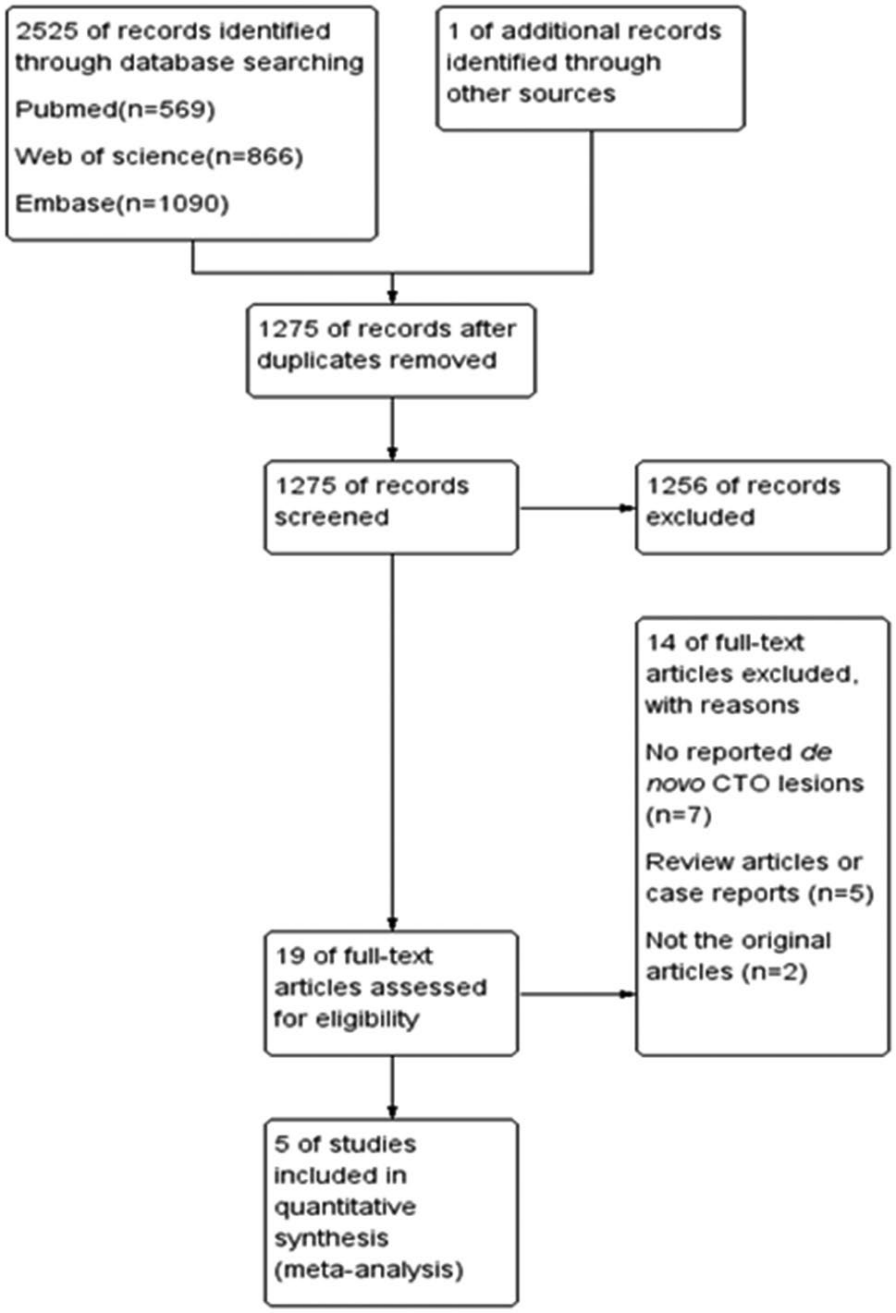
Flow diagram of the study selection process

The initial search generated 2525 potentially relevant articles. After removing duplicate records and preliminary screening, 19 articles were included in the meta-analysis through full text evaluation. Studies were then excluded for one of the following reasons: (a) they did not report de novo CTO lesions; (b) they were not original articles; and (c) they were review articles or case reports. Finally, a total of 5 studies were available for the meta-analysis, including 511 patients [11–15]. Figure 1 shows the flowchart of the study selection procedure.

The sample sizes of the selected studies varied from 34 to 281 patients. The baseline data and characteristics of the included studies are summarized in Table 1. Across studies, patients were predominantly male (72.9-85.7%) and over fifty years old (Table 1).

**Table 1:** Main characteristics of the included studies in the meta-analysis

References	Date	Number of patients	Male (%)	Age	Mean DCB length (mm)	Mean DCB diameter (mm)
Wickramarachchi et al.	2017	41	85.4	62.1 ± 9.5	41 ± 26	2.8 ± 0.6
Köln et al.	2016	34	76.5	59.2 ± 12.8	50.42 ± 18.47	2.55 ± 0.42
Terashita et al.	2023	71	76.1	67.7 ± 11.2	47.1 ± 19.7	2.78 ± 0.43
Jun et al.	2022	84	85.7	56.1 ± 9.9	42.3 ± 17.1	2.7 ± 0.4
Wang et al.	2023	281	72.9	58.8 ± 10.9	35.81 ± 19.92	2.63 ± 0.38

DCB: drug-coated balloon

### Primary outcomes

**Fig. 2:**
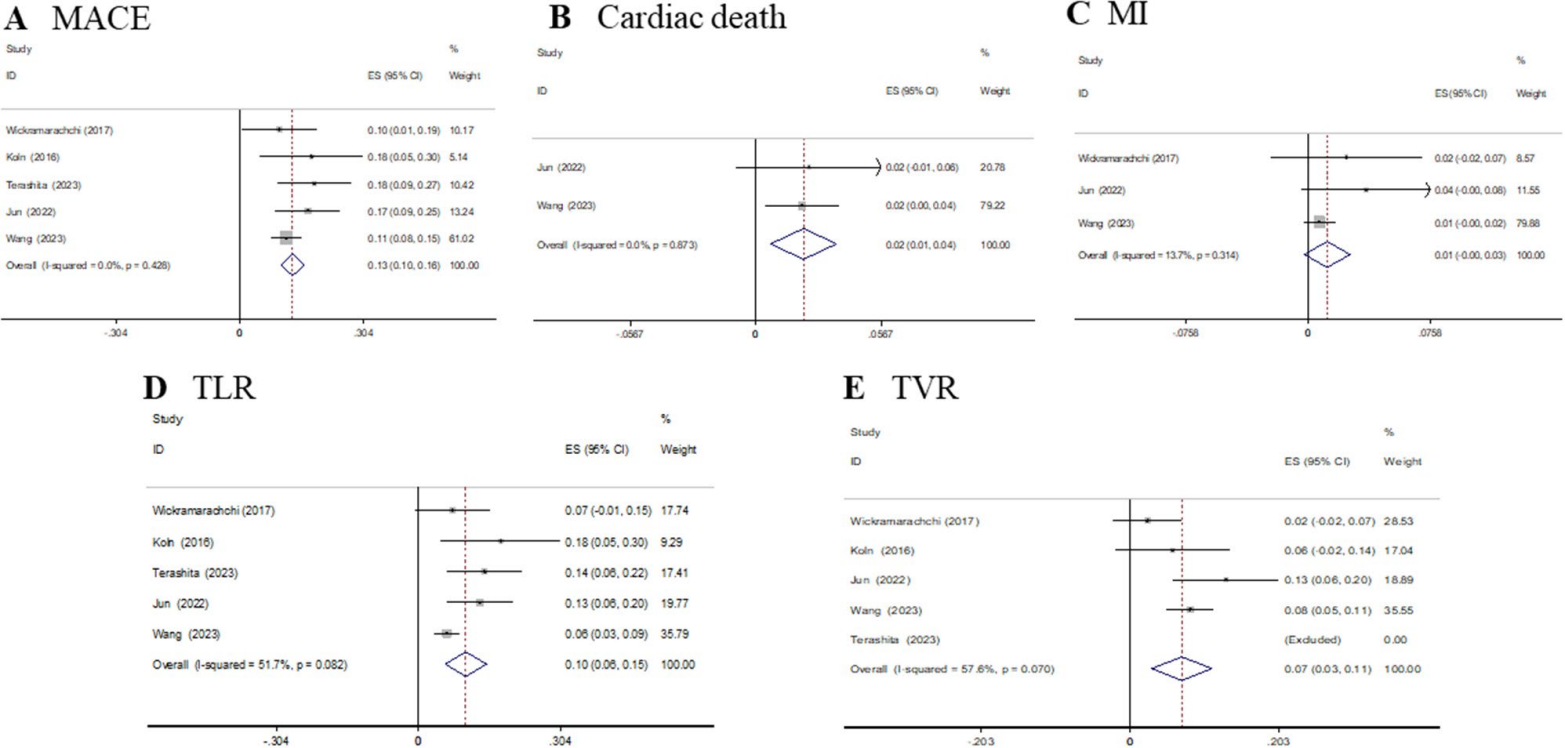
Forest plots of incident complications after DCB treatment in CTO patients

The primary analysis on the composite endpoint of MACE including all results of the studies is presented in Table 2. The summary estimate rate of MACE was 13.0% (95% CI 10.1%-15.9%, I^2^ = 0%, *p* = 0.428). The summary estimate rates of cardiac death and MI were 2.2% (95% CI 0.7%-3.7%, I^2^ = 0%, *p* = 0.873) and 1.2% (95% CI -0.2-2.6%, I^2^ = 13.7%, *p* = 0.314), respectively. Finally, the pooled incidences of TLR and TVR were 10.1% (95% CI 5.7%-14.5%, I^2^ = 51.7%, *p* = 0.082) and 7.1% (95% CI 3.0%-11.2%, I^2^ = 57.6%, *p* = 0.070), respectively (Fig. 2).

**Table 2:** Complications of included studies in the meta-analysis

References	Follow up time	MACE (%)	Cardiac death (%)	MI (%)	TLR (%)	TVR (%)
Wickramarachchi et al.	313 (304) days	9.8	-	2.4	7.3	2.4
Köln et al.	8.62 ± 9.33 months	17.6	-	-	17.6	5.8
Terashita et al.	29 (13-51months)	18.3	-	-	14.1	-
Jun et al.	720 (406-1,268) days	16.7	2.4	3.6	13.1	13.1
Wang et al.	3 years	11.4	2.1	0.7	6.0	8.2

MACEs: major advent cardiovascular events; MI: myocardial infarction; TLR: target lesion revascularization; TVR: target vessel revascularization

**Fig. 3:**
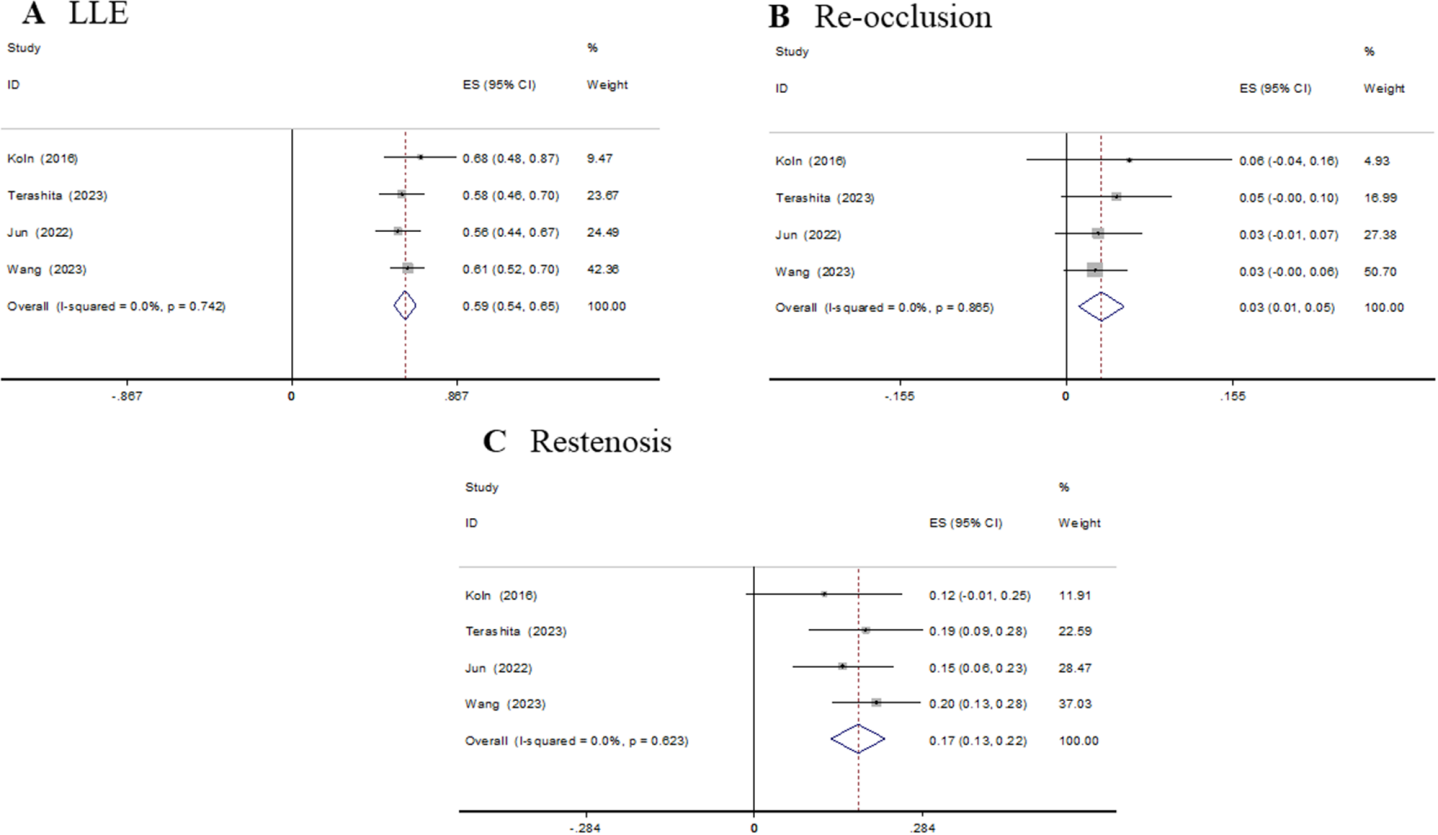
Forest plots of follow-up angiographic endpoints after DCB treatment in CTO patients

Four studies reported LLE, reocclusion and restenosis. The follow-up angiographic endpoints are reported in Table 3. The summary estimate rates of LLE, reocclusion and restenosis were 59.4% (95% CI 53.5–65.3%), 3.3% (95% CI 1.1–5.4%) and 17.5% (95% CI 12.9–22.0%), respectively. There was significant heterogeneity in the estimation of angiographic endpoints, with I^2^ ranging from 62.3 to 86.5%. Thus, the random effects model was used for the meta-analysis (Fig. 3).

**Table 3:** The follow-up angiographic endpoints of the included studies in the meta-analysis

References	Follow-up angiography		
	LLE (%)	Reocclusion (%)	Restenosis (%)
Köln et al.	67.6	5.9	11.8
Terashita et al.	57.8	5.7	18.8
Jun et al.	55.6	3.0	14.9
Wang et al.	60.7	2.7	20.5

LLE: late lumen enlargement

## Discussion

CTO represents one of the most challenging procedures in PCI, accounts for approximately 20% of selective coronary angiography [1]. With advancements in CTO recanalization intervention technology and concepts, the success rate of recanalization continues to increase [4]. Compared to optimal medical therapy, successful PCI revascularization can effectively alleviate clinical symptoms related to myocardial ischemia and improve prognosis [3]. Although the newer-generation of DESs significantly reduce the incidence of restenosis and clinical adverse events, they still pose risks such as atherosclerosis due to their proinflammatory effect, stent thrombosis and stent malapposition [16]. Diffuse lesions in CTO typically require multiple stent or long stent implantation, which increases the risk of stent-related adverse events. Compared with DES, DCB can mitigate these risks, and in the absence of foreign implantation, short-term elution drugs can effectively prevent endometrial hyperplasia, which has been confirmed in several long-term follow-up DCB experiments [17, 18].

Initially, DCB was originally used to treat coronary ISR after BMS implantation [19]. Meanwhile, there is increasing evidence of the use of DCBs for the treatment of bifurcation and small vessel lesions [20]. Previous studies indicated that in small coronary lesions treated with DCB, the target lesion thrombosis rate was 0.6% at six months, with no event occurred during the final follow-up period of six months to three years [17]. The cell-inhibitory drugs delivered by DCB are released into the vasculature over a short period, contrasting with the six to twelve-month drug release period of DES. DCB has a broader and more homogeneous distribution of drugs in the vascular lumen than DES, which has a particularly beneficial effect in treating plaque-filled CTO lesions. We believe that successful recanalization of CTO lesions using DCB treatment can overcome the inherent drawbacks of stent implantation.

Our meta-analysis demonstrated promising outcomes for DCB treatment in CTO-PCI, showing a lower major adverse cardiovascular event (MACE) rate of 13.0%. In the DECISION-CTO trial [21], the MCAE (death, MI, stroke, or any revascularization) of the CTO-PCI group (receiving DES implantation) at three years was 21.5%, which was greater than our results. In addition, the incidence of cardiac death and TVR in DECISION-COTRIAL was 1.9% and 7.9%, respectively [21], This study showed comparable event occurrence rates (2.2% and 7.1%, respectively). However, as DECISION-COTRIAL is a single clinical trial, more studies are needed to compare the above data due to possible differences in study design, patient characteristics and treatment strategies.

In our study, the pooled rates of reocclusion and restenosis were 3.3% and 17.5%, respectively, among patients who underwent angiography follow-up. In the study by Onishi et al. to validate the efficacy of DCB treatment in de novo coronary artery lesions, including bifurcation and CTO lesions, the restenosis rate in the CTO lesion subgroup was 17% at 8.2 ± 4.0 months of follow-up after PCI [22], which was comparable to our results. Valenti et al. demonstrated that the restenosis rate was similar (12.5%) in CTO patients receiving DES treatment [23]. The risk of restenosis and stent thrombosis in CTO patients remains a challenging problem. One of the mechanisms is that stents are common in newly opened CTOs, and their size is too small because blood vessels do not immediately expand [24]. Due to the lack of blood flow for a long time, the distal vessel of CTO is narrowed. After balloon angioplasty, antegrade blood flow increases; however, it may take several weeks or months for blood vessels to dilate. Therefore, after CTO balloon angioplasty, it is easy to underestimate the actual size of the blood vessel, which increases the risk of inadequate stent size, poor stent adherence, and stent thrombosis [12]. Conversely, after DCB treatment, the blood vessels may recover to their original size in the future, without the need for stents to fix the size of the vessels. This is one of the most attractive and important advantages of using DCB to treat CTO lesions.

One of the main benefits of DCB in treating CTO lesions is the possibility of vascular remodeling over time. Previous reports have shown that 50-70% of non-CTO lesions treated with DCB exhibit LLE upon follow-up angiography and intravascular imaging [25, 26]. Scheller et al. first reported on LLE after DCB angiography in de novo coronary artery lesions and subsequently published a study using quantitative coronary angiography (QCA) analysis to evaluate LLE after DCB angioplasty [27]. After an average follow-up of 4.1 ± 2.1 months, 69% of patients developed LLE [28]. In our study, the summary estimate rate of LLE was 59.4% for CTO lesions. The possible mechanism of the enlargement of the vascular lumen likely involves apoptosis of smooth muscle cells (SMCs), as paclitaxel inhibits SMC proliferation and promotes proapoptotic factors through microtubule regulation [29]. Higher initial paclitaxel concentrations post-DCB compared to paclitaxel-eluting stents lead to mitotic arrest. Meanwhile, it has been confirmed in animal models that local application of paclitaxel can inhibit neointimal growth and facilitate arterial dilation [30]. However, research has shown that intracellular microtubules are specific binding sites for paclitaxel, mainly present in the subintima and adventitia [31]. Therefore, the retention of paclitaxel in these layers is significantly higher. when using DCB for subintimal recanalization, the local paclitaxel concentration may exceed the therapeutic effect within the toxic range, promoting excessive dilation of the vascular wall after DCB angioplasty, leading to coronary artery aneurysm. Another possible explanation of the enlargement of the vascular lumen might be arterial healing. Post-recanalization, the previously occluded segment undergoes extravascular remodeling due to long-term hypoperfusion. Over time, the restoration of anterograde blood flow and perfusion pressure helps to return the segment to its original size [13]. In addition, considering that most CTO segments contain organized thrombi rich in fibrin or proteoglycans in their occluded segments, the recovery of blood flow may promote the dissolution of organized thrombi, thereby further increasing the lumen [32]. Patients undergoing PCI must require daily dual antiplatelet therapy (DAPT) to prevent complications caused by thrombotic events. Another potential benefit is the shorter duration of DAPT and lower risk of bleeding events after DCB treatment, which is particularly important for patients with increased bleeding risk, measured by the CRUSADE score, or planning surgery shortly after PCI.

Detailed preoperative preparation and meticulous CTO PCI surgery are vital to reducing CTO vascular reconstruction and stent implantation [19]. The preparation of lesions has always been emphasized in DCB angioplasty to achieve optimal results [33], especially in CTO lesions. Adequate lesion preparation can increase the contact area between the surface of DCB and the endometrium, and local dissection without blood flow restriction can facilitate the delivery of antiproliferative drugs. Additionally, the combination of DES and DCB is an acceptable choice for the treatment of diffuse coronary lesions, reducing the risk of stent-related events by reducing stent use [34]. As is well known, the longer the stent length is, the higher the incidence of MACE. Meanwhile, DCB provides a “non-implantation” intervention option for cases of bifurcation or small vessel lesions in CTO recanalization to avoid imprisonment or small vessel cage formation caused by DES.

These ongoing trials related to DCB versus DES for treatment of CTO are crucial for advancing our understanding of the comparative effectiveness of DCB versus DES in the management of chronic total occlusions. By evaluating outcomes such as procedural success rates, recurrence rates, and long-term clinical outcomes, these trials can provide valuable insights into the optimal treatment strategies for patients with CTO. For patients who use DCB-only to treat CTO, especially those with complex features, more research is needed to explore the best revascularization strategies to ensure the best clinical outcomes. In summary, the current data seem to support the potential of DCB as a viable treatment option for suitable CTO patients.

## Limitations

This study had several limitations. First, the number of studies on DCB treatment in CTO is limited, with most being single-center, small sample size studies. Larger-scale and multicenter studies are needed to confirm our findings. Second, most of the studies are observational studies, and more randomized controlled studies on the comparative efficacy of DCB and DES after CTO revascularization are needed in the future. Third, publication bias may affect the results of our meta-analysis, as centers with more experience and higher surgical volumes are more likely to report their outcomes.

## Conclusion

The current data seem to support the use of DCBs in CTO revascularization. DCB may be reasonable when balloon angioplasty results with angiography and endovascular imaging are satisfactory.

### Electronic supplementary material

Below is the link to the electronic supplementary material.

Supplementary Material 1

## Data Availability

The dataset analyzed during the current study are available from the corresponding author on reasonable request.
