# Supplementary material for: Efficacy and safety of drug-coated balloons in chronic total coronary occlusion recanalization: a systematic review and meta-analysis
Source: BMC Cardiovasc Disord. 2024 Jun 26;24:324. doi: 10.1186/s12872-024-03993-x (PMC11200994; doi:10.1186/s12872-024-03993-x)
Supplement: Supplementary file 1 — Supplementary Material 1 [file 12872_2024_3993_MOESM1_ESM.docx]

**Supplementary Table 1 The quality of the included studies.**

| References | Date | Selection | Comparability | Outcome | Nos Score |
| --- | --- | --- | --- | --- | --- |
| Wickramarachchi et al. | 2017 | 4 | 1 | 2 | 7 |
| Köln et al. | 2016 | 4 | 1 | 1 | 6 |
| Terashita et al. | 2023 | 4 | 2 | 2 | 8 |
| Jun et al. | 2022 | 4 | 1 | 2 | 7 |
| Wang et al. | 2023 | 4 | 2 | 2 | 8 |

Nos: the **Newcastle-Ottawa Scale.**
